# Robust screening of atrial fibrillation with distribution classification

**DOI:** 10.1038/s41598-025-10090-2

**Published:** 2025-07-22

**Authors:** Pierre-François Massiani, Lukas Haverbeck, Claas Thesing, Friedrich Solowjow, Marlo Verket, Matthias Daniel Zink, Katharina Schütt, Dirk Müller-Wieland, Nikolaus Marx, Sebastian Trimpe

**Affiliations:** 1 Institute for Data Science in Mechanical Engineering, RWTH Aachen University, Aachen, Germany; 2 Department of Internal Medicine I, University Hospital RWTH Aachen, Aachen, Germany

**Keywords:** Atrial fibrillation, Screening, Support vector machines, Distribution classification, Machine learning, Population screening, Applied mathematics, Computer science, Atrial fibrillation

## Abstract

Atrial fibrillation (AF) correlates with an increased risk of all-cause mortality or stroke, mainly due to undiagnosed patients and undertreatment. Its *screening* is thus a key challenge, for which machine learning methods hold the promise of cheaper and faster campaigns. The *robustness* of such methods to varying artifacts, noise, and conditions is then crucial. We introduce the first distributional support vector machine (SVM) for robust detection of AF from short, noisy electrocardiograms. It achieves state-of-the-art performance and unprecedented robustness on the screening problem while only leveraging one interpretable feature and little training data. We illustrate these advantages by evaluating on other data sources (*cross-data-set*) and through sensitivity studies. These strengths result from two main components: (i) preliminary peak detection enabling robust computation of medically relevant features; and (ii) a mathematically principled way of aggregating those features to compare their *full distributions*. This establishes our algorithm as a relevant candidate for screening campaigns.

## Introduction

Atrial fibrillation (AF) is the most widespread cardiac arrhythmia, with a prevalence estimated at $3\%$ in adults^1^. Although it is not, in most cases, a life-threatening condition directly, it correlates with an increased risk of all-cause mortality and of stroke^2^. A core stake of AF is thus its early detection to enable preemptive care and prophylactic treatments, thereby mitigating the risk of aggravated conditions and reducing public health burdens^1^. Yet, a significant portion of the population living with AF remains undiagnosed, primarily those with subclinical AF^2^ even though this condition can be identified from a single-lead electrocardiogram (ECG). Therefore, efficient and broadly accessible screening for early AF detection has been identified as crucial in reducing AF-related complications^1^.

Automated methods together with low-cost sensors hold the potential for large-scale screening campaigns for AF. They can assist physicians by an automatic and fast analysis of patient data, increasing throughput. However, this requires methods with unprecedented *robustness*, as low-cost sensors are notoriously noisy and the populations on which screening algorithms are used often differ statistically from those on which they were calibrated. This effect is known as *distribution shift*.

Methods for automatic AF detection can be broadly classified as being either rule-based^3–9^ or learning-based^10–23^. The former are historically the most widespread due to their simplicity, low computational cost, and interpretability. They are also relatively robust, as they rely on established biomarkers. On the other hand, they can fail to distinguish certain conditions due to the limited number of features they use, and increasing this number makes them difficult to tune. In contrast, learning-based methods hold the promise of increased performance, by allowing more features—some learned from data—and tuning their relative weights and decision thresholds based on examples. While numerous recent studies highlight the excellent performance of such methods, the question of their robustness is much less explored. In fact, recent evidence points to the fact that many methods lack such robustness^13,14^, which is a clear hindrance to their deployment in screening campaigns.

In this study, we propose an algorithm for the robust screening of AF from single-lead ECGs. We find that our method is more sensitive than tested alternatives^24,25^, which is an essential quality for screening. Further, a methodological contribution of our work is the systematic *cross-data-set* evaluation of algorithms, meaning that we evaluate performance on test sets from a source different that that of the training set. We use the DiagnoStick^25^, SPH^26^, and CinC 2017^24^ data sets, as they have varied lengths and noise levels, and do not use the MIT-BIH data set^27^ as its high-quality recordings are not representative of the situation of screening. This enables us to evaluate robustness, an aspect that other studies typically neglect^13^. In fact, we find in our comparative study that the performances of almost all top-performers of the Computing in Cardiology competition^24^ collapse cross-data-set. In contrast, we achieve our robust performance by relying on a *single* feature; namely, R–R intervals (RRis). This strongly supports that it is important for screening to rely on few, but medically-relevant features with high signal-to-noise ratio so that even low-cost sensors can capture them reliably. Such features then need to be exploited to their maximum, requiring a powerful *representation* of their distribution. While the classical approach consists of computing empirical moments in a window of fixed size, we leverage a novel class of algorithms called *distributional support vector machines* (SVMs)^28,29^ that operate on and compare probability distributions directly. This allows them to work with inputs of arbitrary size, refining the estimates of the distributions as more samples become available, which is essential to allow different data sources and, thus, for screening. Furthermore, our SVM is competitive with recent neural networks to which we compare, achieving marginally lower accuracy but higher robustness and sensitivity. Our work thus demonstrates that excellent performance for screening can be achieved with simpler and more interpretable algorithms such as distributional SVMs, contrasting with previous studies^14^ where a clear supremacy of neural networks emerged, as seen by comparing the reported performances of neural networks^10–16^ and of SVMs^14,17–20^. Finally, this work is the first implementation of distributional SVMs to our knowledge, which is then a methodological contribution.

## Results

**Fig. 1:**
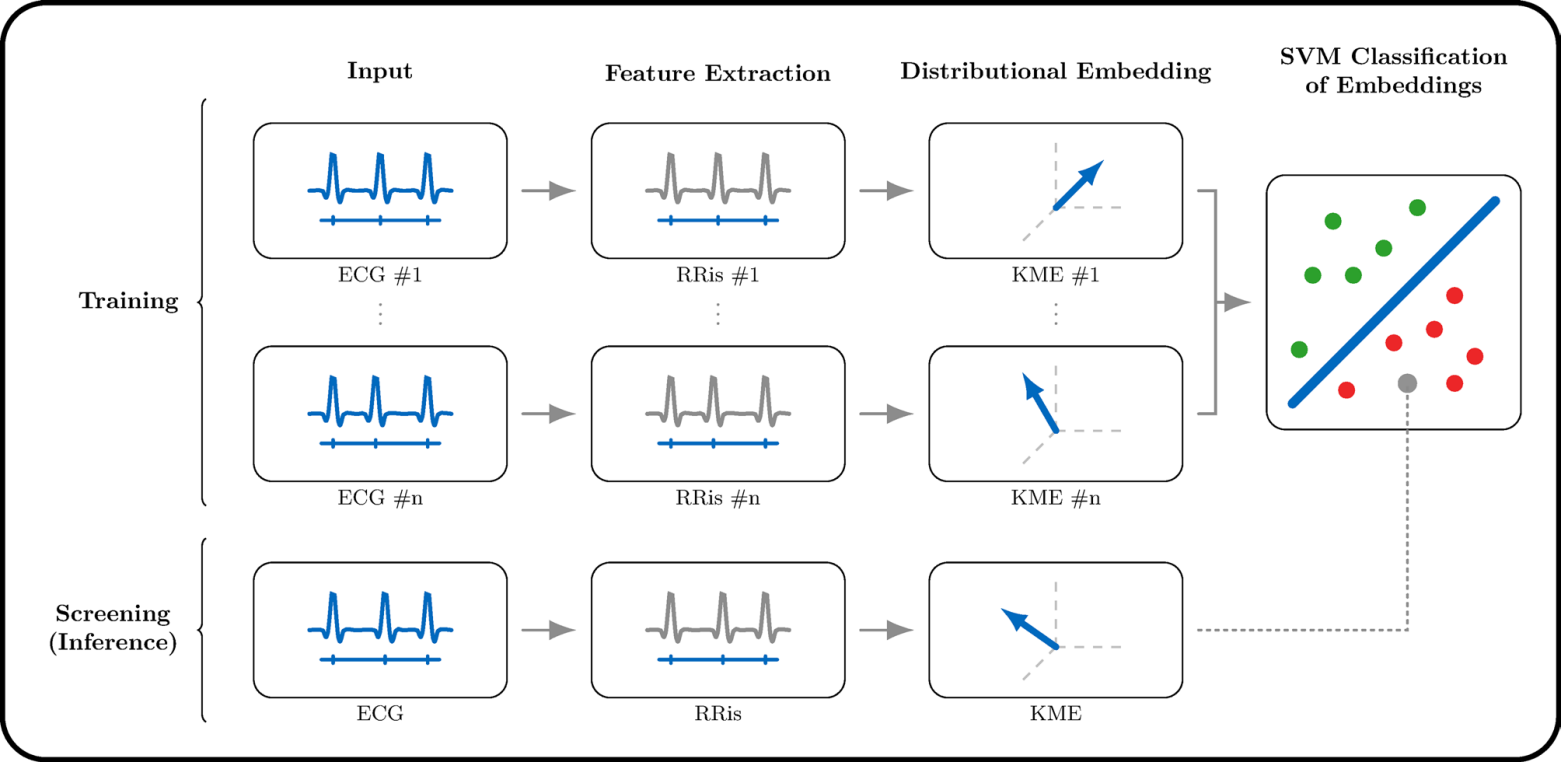
The summary of our method, an SVM for distribution classification. It classifies a new, unseen patient by comparing the *full empirical distribution* of its RRis to those of the training set, enhancing robustness and adaptability to inputs of varying sizes.

We propose the first implementation of SVMs for distribution classification to detect the presence of AF from a short ECG. We evaluate our model on three data sets: MyDiagnoStick^25^, SPH^26^, and CinC 2017^24^ data sets. Their sizes are in Table 1. The exact length of the ECG depends on the specific data set at hand and may vary between training and inference time. We remind the reader that “in-data-set” refers to the configuration where both the training and testing set are from the same original data set, while “cross-data-set” to that where they are from different data sets. The model takes as an input the outcome of a preliminary peak detection (Fig. 1).

**Table 1:** Set sizes.

Set	Training	Validation	Testing
DiagnoStick	4326	1442	1441
SPH	6364	2121	2120
CinC	5117	1706	1705

### Performance and cross-data-set stability

**Fig. 2:**
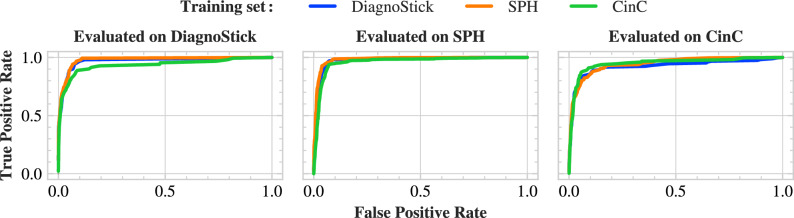
Cross-data-set ROC curves.

We report accuracy, F1-score, sensitivity, and specificity, respectively defined as

$$\begin{aligned} \text {Accuracy}&= \frac{\text {TP}+ \text {TN}}{N},&\text {F}1\text {-Score}&=\frac{2\text {TP}}{2\text {TP}+ \text {FP}+ \text {FN}},\\ \text {Sensitivity}&= \frac{\text {TP}}{\text {TP}+ \text {FN}},&\text {Specificity}&=\frac{\text {TN}}{\text {TN}+\text {FP}}, \end{aligned}$$

where *N* is the number of samples and $\text {TP}$, $\text {TN}$, $\text {FP}$, and $\text {FN}$ are respectively the numbers of true positives, true negatives, false positives, false negatives. We also report area under the receiving operator characteristic (AUROC), and provide the corresponding ROC curves (Fig. 2). We achieve excellent performance in-data-set; for comparison, we outperform both the proprietary algorithm of the MyDiagnoStick medical device and the neural network and SVM of^14^ on the DiagnoStick and SPH data sets, respectively. The only exception is the CinC data set, where we have lower F1-score than top-performers of the competition^24^. Furthermore, our performance is robust cross-data-set; for each test set, the attained F1-score only fluctuates by at most 2 points upon switching the training set, with the exception of the SPH data set where in-data-set training yields better results. This is not the case for the baselines, whose performance all collapse cross-data-set, with the notable exception of^30^ which achieves similar performance as our method but a moderately inferior sensitivity. This stability across different training sets demonstrates the robustness of our method, as the classification performance is only mildly affected by this distribution shift between training and inference. Finally, a visual inspection of the misclassified examples of the DiagnoStick test set highlights that peak extraction is of poor quality on those examples (Supplementary Table S1).

**Fig. 3:**
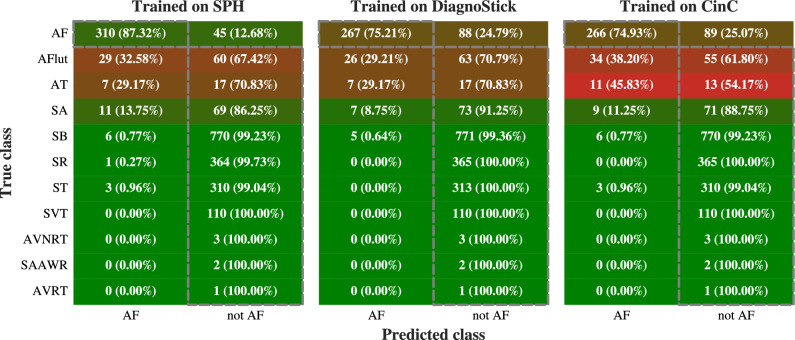
Confusion matrices of the classifier trained on different data sets and evaluated on the SPH data set. True positives and negatives are circled in gray. Acronyms on the *y*-axis are those defined in the documentation of the SPH data set^26^, with the exception of atrial flutter and atrial fibrillation, which we rename as “AFlut” and “AF” instead of “AF” and “AFib”, respectively. In short, AF is atrial fibrillation, SR is normal sinus rhythm, and other labels are conditions other than AF. We see that training cross-data-set on the DiagnoStick or CinC data set only marginally changes the matrix, indicating that most examples are classified identically and showing robustness. Further, normal sinus rhythm is almost perfectly separated from AF.

Confusion matrices (Fig. 3) reveal that the confusions are mainly independent of the training set and arise from conditions that are hard to tell apart from AF purely based on RRi, such as sinus arrhythmia (SA) and atrial flutter (AFlut). Normal sinus rhythm, however, is almost perfectly separated.

### Data efficiency

**Fig. 4:**
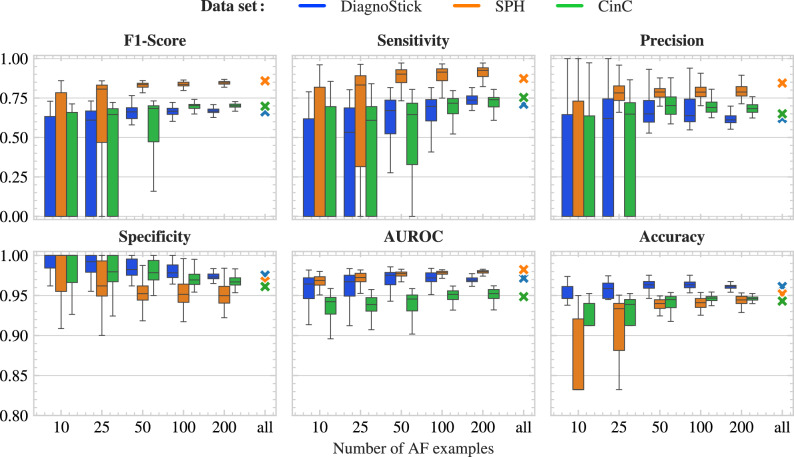
Testing performance with increasing training set size. Each box plot is obtained with 100 independent resamplings of the training set.

We examine the data efficiency of our algorithm by training it on sets of increasing sizes (Fig. 4). Each training is repeated several times to account for the non-deterministic choice of the training set. The results show excellent and consistent performance is already reached with training sets containing about 50 to 100 positive examples.

**Table 2:** Performance metrics on test sets, in percentage points, for our method, the proprietary algorithm, and the baselines^30–33^.

Algorithm	Training	Testing	Acc.	F1	Sen.	Spe.	AUROC	Noise
Ours	DiagnoStick	DiagnoStick	96.12	66.26	71.05	97.54	97.17	–
Ours	SPH	DiagnoStick	95.76	66.29	77.63	96.79	98.20	–
Ours	CinC	DiagnoStick	96.68	67.13	63.16	98.58	93.49	–
Ours	DiagnoStick	SPH	93.72	80.06	75.21	97.45	97.56	–
Ours	SPH	SPH	95.18	85.87	87.32	96.77	98.24	–
Ours	CinC	SPH	92.82	77.78	74.93	96.43	96.11	–
Ours	DiagnoStick	CinC	93.22	66.67	77.54	94.73	92.44	–
Ours	SPH	CinC	92.72	65.88	80.43	93.89	94.48	–
Ours	CinC	CinC	94.30	69.80	75.36	96.11	94.85	–
Proprietary	–	DiagnoStick	92.51	50.93	72.37	93.65	–	–
Datta et al.^30^	$\hbox {CinC}^\star$	DiagnoStick	96.96	66.14	55.26	99.33	–	1.27
Hong et al.^31^	$\hbox {CinC}^\star$	DiagnoStick	94.63	0.00	0.00	100.00	–	84.53
Zabihi et al.^32^	$\hbox {CinC}^\star$	DiagnoStick	96.33	54.39	40.79	99.48	–	4.87
Mahajan et al.^33^	$\hbox {CinC}^\star$	DiagnoStick	95.34	37.74	26.32	99.25	–	11.65

The classifiers of^30–33^ were trained during the CinC 2017 competition^24^, which we indicated as the training set “$\hbox {CinC}^\star$”. We refer to the individual references for details. The abbreviations in the headers of the columns 4–7 stand for, “Accuracy”, “F1-Score”, “Sensitivity”, and “Specificity”, respectively. The “Noise” column indicates the proportion of ECGs classified as noise by the algorithms of the CinC 2017^24^ competition. They are counted as negative classifications for AF presence.

### Influence of peak detection

We run our pipeline for different peak detection algorithms on all validation data sets and find that the performance highly depends on the choice of the algorithm (Table 3). Moreover, some algorithms perform particularly poorly on noisy data sets. The XQRS algorithm^34^ outperforms or is equally as good as all others on all data sets, and is the one that we implement for the rest of the results.

**Table 3:** In-data-set F1-score, in percentage points, on validation data sets.

Algorithm	DiagnoStick	SPH	CinC
XQRS^34^	$\mathbf {75.83}\ (\pm 2.23)$	$\mathbf {85.05}\ (\pm 1.24)$	$\mathbf {69.23}\ (\pm 2.75)$
Christov^35^	$52.81\ (\pm 7.08)$	$78.93\ (\pm 1.47)$	$55.29\ (\pm 7.60)$
Elgendi et al.^36^	$63.04\ (\pm 1.97)$	$74.05\ (\pm 2.40)$	$20.16\ (\pm 4.99)$
Hamilton^37^	$73.26\ (\pm 5.45)$	$76.26\ (\pm 1.73)$	$63.90\ (\pm 4.05)$
Rodrigues et al.^38^	$73.80\ (\pm 2.03)$	$84.06\ (\pm 1.16)$	$67.19\ (\pm 2.54)$
Zong et al.^39^	$18.80\ (\pm 4.94)$	$71.79\ (\pm 1.41)$	$52.92\ (\pm 2.62)$
Makowski et al.^40^	$73.54\ (\pm 3.77)$	$84.99\ (\pm 2.08)$	$64.51\ (\pm 4.46)$
Pan and Tompkins^41^	$71.68\ (\pm 3.35)$	$80.33\ (\pm 1.12)$	$65.02\ (\pm 4.53)$

Bold font indicates the best of each column. The scores are the means over the held-out fold during cross-validation, and the value in parentheses is the variance over that fold. The main results of Table 2 use the XQRS algorithm.

## Discussion

Our method achieves state-of-the-art performance on the SPH and DiagnoStick data sets, outperforming the proprietary algorithm of the MyDiagnostick medical device and the SVM and neural network of^14^. It is not directly competitive^1^ in-data-set with the top-performers of the CinC 2017 competition^24^, but it significantly outperforms them cross-data-set, demonstrating unprecedented robustness to distribution shift (with the exception of^30^, which is also robust). Furthermore, our method is the most sensitive of all evaluated alternatives.

A first conclusion our results support is that SVMs can be competitive with neural networks for AF screening—an observation that deviates from recent results on learning-based detection of AF. This is particularly important for deployment, as SVMs based on well-understood features are notriously more interpretable than black-box neural networks trained end-to-end. The essential component enabling this improvement is the distributional embedding step of our classifier. It extracts more information from this feature than a classical SVM would and bases the classification decision on the *full distribution* of the feature, as opposed to only using a few moments. This step also provides a principled and theory-backed answer to a recurring challenge of cross-data-set evaluation; namely, adapting the size of the input to match the expectations of the classifier. Indeed, our distributional SVM works with inputs of arbitrary size, possibly differing from that seen at training.

A second conclusion is that it can be beneficial for screening to rely on few, but medically-relevant prominent features that are exploited to their maximum rather than to multiply statistical features. Indeed, this provides the resulting classifiers with inherent robustness and data-efficiency, as even low-quality sensors are able to capture them reliably and sensor- or population-specific statistical artifacts are filtered out. It is clear that classifiers relying on such few features may not separate conditions that require additional information to be told apart; yet, this is not necessarily problematic for screening, where false positives are preferred over false negatives to an extent and the gained robustness and data efficiency can be essential.

The robustness of our classifier is largely influenced by that of the peak detection algorithm it uses. We find high variability in our results depending on the choice of such an algorithm, despite all of them reporting excellent performance on their respective benchmarks. An examination of our classifier’s mistakes on the DiagnoStick data set (supplementary material) suggests that misclassifications mainly occur together with inaccurate peak detection, which is in turn primarily due to noise. This highlights the crucial role of these algorithms for robust and interpretable AF detection, as they enable extracting the features effectively used by practitioners. Perhaps a part of the effort on end-to-end AF detection could thus be redirected to extracting these features reliably when existing algorithms fail. Such a pipeline including separate feature extraction has already shown promising results for robustness in cancer mutation prediction ^42^, and our results support that this robustness carries to AF detection.

## Methods

### Data sets

The DiagnoStick data set consists of 7209 1 min-long ECGs recorded at a frequency of $200\,\textrm{Hz}$ with the MyDiagnostick medical device (hand-held, single-lead ECG device)^25^. The data was collected in pharmacies in Aachen for research assessing the efficiency of the MyDiagnoStick medical device. Each ECG comes with a binary label indicating whether or not it shows AF. The data set is composed of 383 recordings with AF (labeled “AF” in the remainder of this work), and 6745 recordings without AF (labeled “noAF”). The remaining 81 recordings have a label “Unknown” and are discarded in this study. These labels were determined by medical practitioners, as reported in^25^.

The MyDiagnoStick medical device also comes with a proprietary algorithm that classifies recorded ECGs. We refer to these labels as the “Proprietary” ones; they may differ from the ones outlined above. We use the proprietary labels to compare against this proprietary algorithm.

The SPH data set consists of 10646 recordings of 12-lead ECGs collected for research on various cardiovascular diseases^26^. Each recording lasts $10\,\textrm{s}$, is sampled at a frequency of $500\,\textrm{Hz}$, and was annotated with one of ten rhythm labels by medical professionals, which include AF. In the original presentation of the SPH data set, the acronym AF refers to atrial flutter. We reserve AF for atrial fibrillation, and denote atrial flutter with AFlut. We only consider the first lead and collapse the annotations to AF and noAF to perform binary classification. In particular, the noAF class includes normal sinus rhythm and other conditions that are not AF.

The CinC data set consists of 8528 recordings of single-lead ECGs, lasting from about $30\,\textrm{s}$ to $60\,\textrm{s}$ and sampled at $300\,\textrm{Hz}$^24^. Each ECG is labeled with one of four labels among “Normal”, “AF”, “Other rhythm”, and “Noisy”. We disregard “Noisy” ECGs, and collapse the labels “Normal” and “Other rhythm” into a class labeled “noAF” to perform binary classification.

We use the denoised version of the SPH data set^26^ and do not apply further filtering. This denoising is a low-level filter available on most ECG acquisition devices^26^. The DiagnoStick and CinC data sets are not further denoised.

The three data sets are independently split into stratified training ($60\%$), validation ($20\%$), and testing ($20\%$) sets. For the SPH and CinC data sets, the stratification is performed before we collapse the labels to “AF” and “noAF”, such that the training, validation, and test sets all have the same proportions of each class with the original labels. A summary of the sizes of the final sets is available in Table 1. All reported results refer to the testing data, which were never used to train or tune the model, with the exception of the results of Table 3 which use the merged data sets between validation and testing.

### Data preprocessing

Each recording is processed by an automatic peak detection algorithm that identifies the time indices of the R-peaks. We then calculate the RRis of each ECG by subtracting these successive time indices. The ECG is then discarded and represented instead by its collection of RRis. Each collection is normalized independently (see below). In what follows, we omit the adjective “normalized” when referring to RRis or their distributions for conciseness. We discard recordings with fewer than 8 (SPH data set), 51 (DiagnoStick data set), or 20 (CinC data set) peaks.

### Feature vector

Let $N\in \mathbb {N}$ be the number of patients, and denote for each $n\in \{1,\dots ,N\}$ the time index of the location of the *i*-th R-peak of patient *n* by $t_i^{(n)}$. The RRis of patient *n* are then $\delta ^{(n)}_i = t_{i+1}^{(n)} - t^{(n)}_i$. We aggregate them in a single vector, which we then normalize to obtain the feature vector

$$\begin{aligned} x^{(n)} = \begin{pmatrix}x_i^{(n)}\end{pmatrix}_{i=1,\dots ,d_n} = \begin{pmatrix}\frac{\delta ^{(n)}_{i}}{\frac{1}{d_n}\sum _{j=1}^{d_n}\delta _{j}^{(n)}}\end{pmatrix}_{i=1,\dots ,d_n}\in \mathbb {R}^{d_n}, \end{aligned}$$

where $d_n$ is the number of intervals available for patient *n*. This normalization enables setting the average value of each feature vector to 1, without units. A data set $\mathcal {D}$ is then a collection of feature vectors with their corresponding labels $\mathcal {D}=\{(x^{(n)}, y^{(n)})\mid n\in \{1,\dots ,N\}\}$, where $y^{(n)}=1$ represents AF and $y^{(n)} = -1$ represents noAF. This corresponds to the step “Feature Extraction” in Fig. 1.

### SVM for distribution classification

The most robust marker to assess AF from an ECG is through the irregularity of the interval between R-peaks^2,43^. In other words, the distribution of RRis is more spread in the presence of AF than without it. The goal of our SVM for distribution classification is to classify whether a patient’s RRis distribution is more similar to that of a patient with or without AF. Unfortunately, we do not have access to that distribution, but only to the samples $x^{(n)}_i$, which we assume to be independent and identically distributed (i.i.d.) according to $P^{(n)}$, the RRis distribution of patient *n*. We explicitly note here that the vectors $x^{(n)}$ may have different lengths, contrary to classical SVMs. We use these i.i.d. samples to approximate a representation of $P^{(n)}$ with its empirical distribution, and then leverage an SVM for classification on this representation. This procedure is called *two-stage sampling*^28,29^.

We now detail the distributional SVM, which builds on ideas and concepts from classical SVMs, of which a complete introduction can be found in ^44^, Chapter 7. We begin with a symmetric, positive semi-definite kernel function $k:\mathbb {R}\times \mathbb {R}\rightarrow \mathbb {R}$. The core idea is to represent each distribution $P^{(n)}$ with its *kernel mean embedding (KME)* $\mu ^{P^{(n)}}$, defined as

1 $$\begin{aligned} \mu ^{P^{(n)}} = \int _{\mathbb {R}} k(\cdot ,x)\textrm{d} P^{(n)}(x). \end{aligned}$$

We would like to run an SVM for binary classification on the set of input-output pairs ${\mathscr {D}}_\mu = \{(\mu ^{P^{(n)}},y^{(n)})\}$. Such an SVM would then require another kernel function $K:{\mathscr {H}}\times {\mathscr {H}}\rightarrow R$, where $K(\mu ^{P^{(n)}},\mu ^{P^{(m)}})$ is the similarity between two kernel mean embeddings $\mu ^{P^{(n)}}$ and $\mu ^{P^{(m)}}$. Here, ${\mathscr {H}}$ is the set in which all of the kernel mean embeddings lie. Unfortunately, we do not have access to $\mu ^{P^{(n)}}$, and are thus unable to compute the corresponding value of *K*. The *second-stage sampling* then consists of approximating $\mu ^{P^{(n)}}$ with its empirical estimator $\mu ^{(n)}$, defined as

2 $$\begin{aligned} \mu ^{(n)} = \frac{1}{d_n}\sum _{i=1}^{d_n} k\left( \cdot ,\,x^{(n)}_i\right) , \end{aligned}$$

and to approximate the Gram matrix of *K* with the approximate Gram matrix $(K(\mu ^{(n)}, \mu ^{(m)}))_{m,n}$. This procedure corresponds to the step “Distributional Embedding” in Fig. 1. Summarizing, the full learning algorithms consists of first performing feature extraction to compute the vector $x^{(n)}$ for each patient, then estimating the KME through (2), and finally performing SVM classification on the empirical KMEs with the kernel *K* (Fig. 1).

This algorithm requires choosing two different kernel functions, *k* and *K*. We use for both a Gaussian kernel,

$$\begin{aligned} k(z_1, z_2) = \exp \left[ -\frac{(z_1 - z_2)^2}{\sigma ^2}\right] ,\quad \text {and}\quad K(\mu _1,\mu _2) = \exp \left[ -\frac{\Vert \mu _1 - \mu _2\Vert ^2_{{\mathscr {H}}}}{2\gamma ^2}\right] \end{aligned}$$

where $\sigma ^2$ and $\gamma ^2$ are hyperparameters whose choices are discussed next, and

3 $$\begin{aligned} \Vert \mu ^{(n)} - \mu ^{(m)}\Vert ^2 = \sum _{i,j=1}^{d_n} k\left( x_i^{(n)},x_{j}^{(n)}\right) + \sum _{i,j=1}^{d_m} k\left( x_i^{(m)},x_{j}^{(m)}\right) - 2\sum _{i=1}^{d_n}\sum _{j}^{d_m} k\left( x_i^{(n)},x_{j}^{(m)}\right) . \end{aligned}$$

We refer to^45^ for more details on (3).

### Hyperparameter tuning

All hyperparameters are selected via in-data-set 5-fold cross-validation on the merged training and validation sets. Specifically, we perform a grid search over $\sigma$, $\gamma$, and the costs of misclassification of the loss function, and choose the combination maximizing the mean AUROC on the held-out fold. We apply this procedure to each candidate peak-extraction algorithm and ultimately select the XQRS algorithm^34^, because it performs the best across data sets (see Table 3). Detailed instructions to reproduce the hyperparameter study are available in the code.

### Baselines

The baselines^30–33^ were selected as the four best-scoring models of the CinC 2017 competition^24^, with the exception of^46^ as the code available on the competition’s website did not run. In particular, we used the published models directly and did not re-train them.

## Supplementary Information

Supplementary Information.

## Data Availability

The SPH data set that supports the findings of this study is available in Figshare with the identifier https://doi.org/10.6084/m9.figshare.c.4560497, and the CinC data set accompanies the publication^24^ and is available on Physionet at the link https://doi.org/10.13026/d3hm-sf11. The DiagnoStick data set was first presented in the article accessible at https://doi.org/10.1007/s00399-020-00711-w and is available from the authors of that article upon request. Alternatively, the DiagnoStick data set can be accessed upon request to Nikolaus Marx.

## Footnotes

1 F1-scores for AF detection of the graded models can be found as the $\textrm{F}1\textrm{a}$ field in the file *results_all_F1_scores_for_each_classification_type.csv* on the competition webpage.

## Code availability

The underlying code for this study is publicly available at https://github.com/Data-Science-in-Mechanical-Engineering/af-detection with instructions on how to reproduce the results and figures.
