# Supplementary material for: Robust screening of atrial fibrillation with distribution classification
Source: Sci Rep. 2025 Jul 22;15:26582. doi: 10.1038/s41598-025-10090-2 (PMC12283955; doi:10.1038/s41598-025-10090-2)
Supplement: Supplementary file 1 — Supplementary Information. [file 41598_2025_10090_MOESM1_ESM.pdf]

Robust Screening of Atrial Fibrillation with Distribution Classification — Supplementary Materials

Pierre-François Massiani[1,*], Lukas Haverbeck[1], Claas Thesing[1], Friedrich Solowjow[1], Marlo Verket[2], Matthias Daniel Zink[2], Katharina Schütt[2], Dirk Müller-Wieland[2], Nikolaus Marx[2], and Sebastian Trimpe[1]

[*]massiani@dsme.rwth-aachen.de
[1]Institute for Data Science in Mechanical Engineering, RWTH Aachen University, Theaterstraße 35–39, Aachen, 52062, Germany
[2]Department of Internal Medicine I, University Hospital RWTH Aachen, Pauwelsstraße 30, Aachen, 52074, Germany

ABSTRACT

This is the supplementary materials to the paper “Robust Screening of Atrial Fibrillation with Distribution Classification”.

Supplementary materials

False Positives	False Negatives
073-PAT-0065, 028-PAT-0084, 041-PAT-0118, 017-PAT-0091, 013-PAT-0016, 078-PAT-0022, 079-PAT-0053, 079-PAT-0056, 064-PAT-0016, 031-PAT-0003, 066-PAT-0051, 057-PAT-0035, 041-PAT-0045, 006-PAT-0034, 058-PAT-0034	090-PAT-0005, 062-PAT-0059, 064-PAT-0067, 029-PAT-0088

Supplementary Table S1. Identifiers of misclassified examples with poorly detected peaks on the DiagnoStick test set, in-data-set. Plots of the corresponding ECGs together with their peaks are available in the next two sections. The identifiers were determined by visual inspection.

Plots of false negatives

This section contains plots of the ECGs of the DiagnoStick data set that were incorrectly classified by our algorithm as not showing the presence of AF. Training was performed on the DiagnoStick data set (in-data-set). The dark blue triangles at the bottom of each plot indicate the times at which the peak detection algorithm (XQRS) detected a peak, and the corresponding peak is highlighted in dark blue in the ECG graph itself.

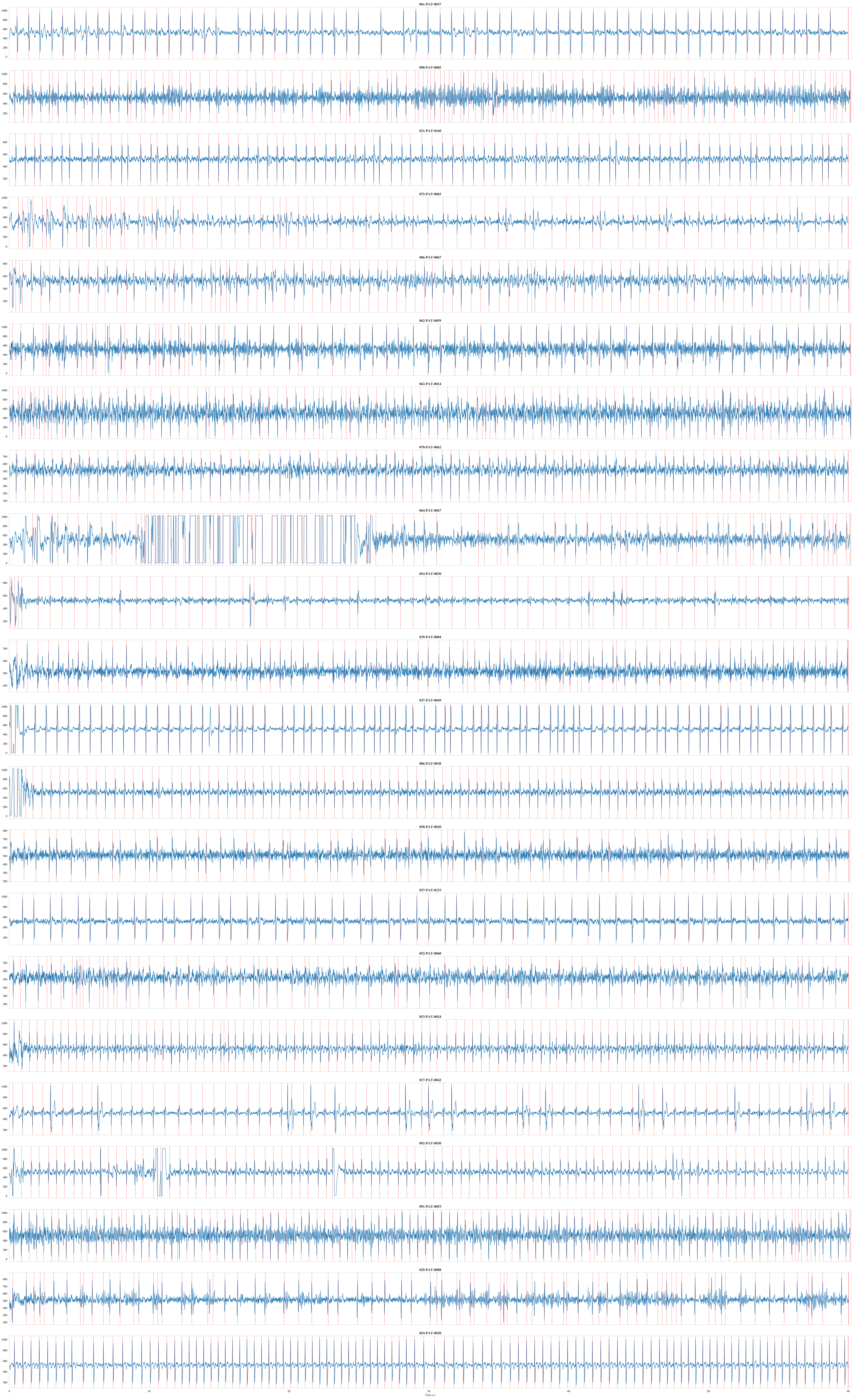
061-PAT-0037
090-PAT-0005
021-PAT-0160
075-PAT-0003
086-PAT-0067
062-PAT-0059
062-PAT-0014
070-PAT-0062
064-PAT-0067
054-PAT-0030
039-PAT-0004
037-PAT-0049
086-PAT-0038
058-PAT-0020
027-PAT-0233
052-PAT-0060
053-PAT-0024
017-PAT-0042
092-PAT-0030
091-PAT-0093
029-PAT-0088
054-PAT-0020
Time (s)

Plots of false positives

This section contains plots of the ECGs of the DiagnoStick data set that were incorrectly classified by our algorithm as showing the presence of AF. Training was performed on the DiagnoStick data set (in-data-set). The dark blue triangles at the bottom of each plot indicate the times at which the peak detection algorithm (XQRS) detected a peak, and the corresponding peak is highlighted in dark blue in the ECG graph itself.

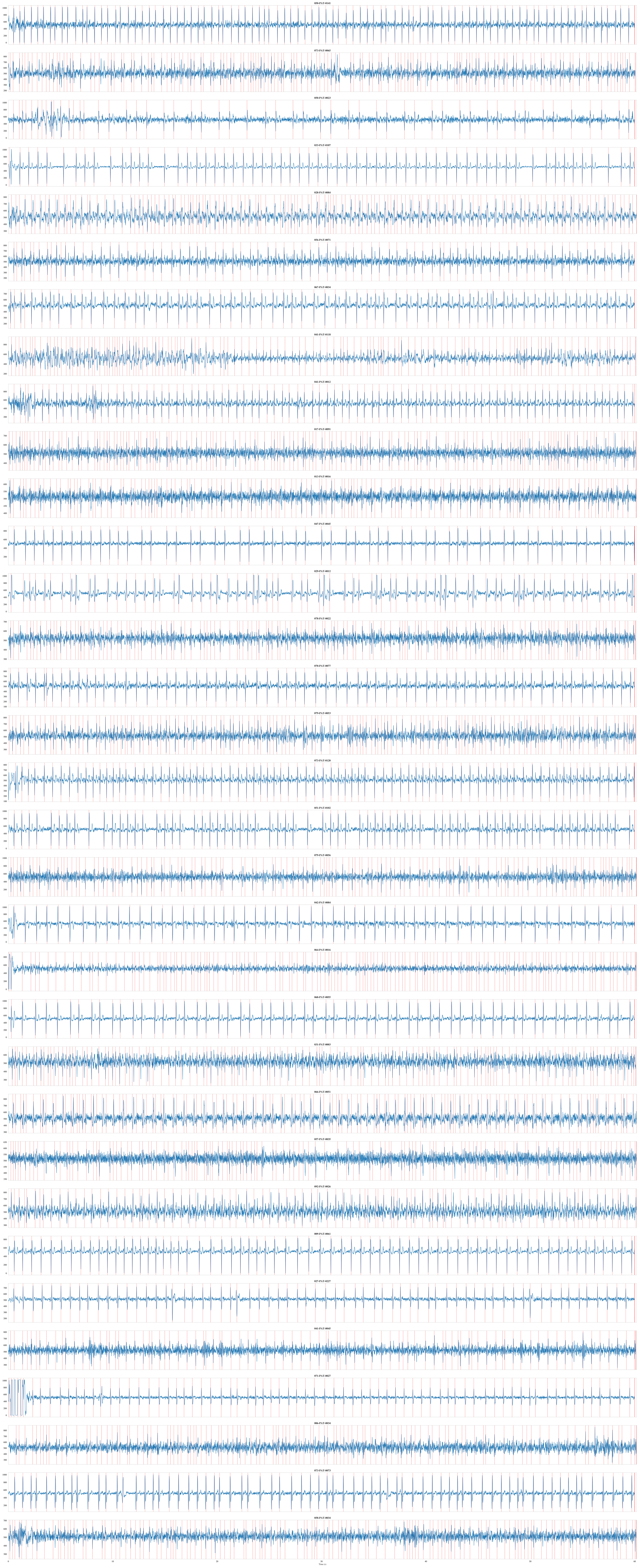
050-PAT-0141
073-PAT-0065
050-PAT-0023
033-PAT-0107
028-PAT-0084
056-PAT-0071
067-PAT-0034
041-PAT-0118
041-PAT-0012
017-PAT-0091
013-PAT-0016
047-PAT-0045
029-PAT-0012
078-PAT-0022
070-PAT-0077
079-PAT-0053
073-PAT-0120
051-PAT-0102
079-PAT-0056
042-PAT-0084
064-PAT-0016
068-PAT-0055
031-PAT-0003
066-PAT-0051
057-PAT-0035
092-PAT-0026
009-PAT-0061
027-PAT-0227
041-PAT-0045
071-PAT-0027
006-PAT-0034
073-PAT-0073
058-PAT-0034
Time (s)
